# Cost-effectiveness and budget impact analysis of screening and preventive interventions for cardiovascular disease in Myanmar: an economic modelling study

**DOI:** 10.1016/j.lansea.2024.100394

**Published:** 2024-04-10

**Authors:** Zin Mar Win, Wenhui Mao, Tom Traill, Zarni Lynn Kyaw, Pyone Yadanar Paing, Osondu Ogbuoji, Gavin Yamey

**Affiliations:** a Community Partners International (CPI), Yangon, Myanmar; b Centre for Policy Impact in Global Health, Duke University, Durham, NC 27708, USA; c Duke Global Health Institute, Duke University, Durham, NC 27708, USA

**Keywords:** Cost-effectiveness analysis, Incremental cost-effectiveness ratio, Budget impact analysis, Cardiovascular disease, Markov model, Myanmar, Screening, Primary and secondary prevention

## Abstract

**Background:**

Cardiovascular diseases (CVD) remains a leading cause of mortality in Myanmar. Despite the burden, CVD preventive services receive low government and donor budgets, which has led to poor CVD outcomes.

**Methods:**

We conducted a cost-effective analysis and a budget impact analysis on CVD prevention strategies recommended by the WHO. A Markov model was used to analyse the cost and quality-adjusted life year (QALY) from healthcare provider and societal perspectives. We calculated transition probabilities from WHO CVD risk data and obtained treatment effects and costs from secondary sources. Subgroup analysis was performed on different sex and age groups. We framed the budget impact analysis from a healthcare provider perspective to assess the affordability of providing CVD preventive care.

**Findings:**

The most cost-effective strategy from the healthcare provider perspective varied. The combination of screening, primary prevention, and secondary prevention (Sc-PP-SP) (incremental cost-effectiveness ratio [ICER]: US$1574/QALY) is most cost-effective at the three times gross domestic product (GDP) per capita threshold, while at one time the GDP per capita threshold, secondary prevention is the most cost-effective strategy (ICER: US$160/QALY). Sc-PP-SP is the most cost-effective strategy from the societal perspective (ICER: US$647/QALY). Among age groups, intervention at age 45 years appeared to be the most cost-effective option for both men and women. The budget impact revealed the Sc-PP-SP would avert 55,000 acute CVD events and 28,000 CVD-related deaths with a cost of US$157 million for the first year of CVD preventive care.

**Interpretation:**

A combination of screening, primary prevention, and secondary prevention is cost-effective to reduce CVD-related deaths in Myanmar. This study provides evidence for the government and development partners to increase investment in and support for CVD prevention. These findings not only provide a basis for efficient resource allocation but also underscore the importance of adopting a total cardiovascular risk approach to CVD prevention, in alignment with global health goals.

**Funding:**

Pilot grant from 10.13039/100006511Duke Global Health Institute, USA.

**Research in context**

**Evidence before this study**

Stroke and ischaemic heart disease are the two leading causes of death in Myanmar. Currently, the healthcare services for cardiovascular disease (CVD) in Myanmar rely heavily on treatment and there is no national screening or prevention program for CVD. High out-of-pocket payments and low awareness of approaches related to CVD risk reduction contributed to the inadequate use of CVD preventive services. We searched PubMed for articles in English up to December 2021 with the following search terms: (“cost effective∗” OR “economic evaluation”) AND cardiovascular∗ AND prevent∗ and there was no cost-effectiveness analysis on CVD in Myanmar. Most studies conducted in neighbouring countries reported that the CVD prevention strategies were cost-effective. A study in Thailand found that primary CVD prevention using a polypill or a combination of three generic blood pressure-lowering drugs was very cost-effective. Another study in Vietnam reported that screening and management strategies for hypertension in CVD prevention were generally cost-effective, though results varied across interventions and age groups.

**Added value of this study**

To our knowledge, this is the first cost-effectiveness analysis and budget impact analysis of CVD prevention strategies for Myanmar. We used a total risk factor approach, and we performed analysis on various preventive interventions including screening, primary, and secondary prevention. We found a combination of screening, primary prevention, and secondary prevention (Sc-PP-SP) would be the most cost-effective strategy from societal perspective at different thresholds. Among age groups, intervention at 45 years appeared to be the most cost-effective option for both men and women. The budget impact revealed the Sc-PP-SP would avert 55,000 acute CVD events and 28,000 CVD-related deaths with a cost of US$157 million for the first year.

**Implications of all the available evidence**

CVD preventive interventions are cost effective in Myanmar and could reduce CVD morbidity and mortality. Our findings generate evidence to inform the government and development partners to increase support for CVD prevention. These findings not only provide a basis for efficient resource allocation but also underscore the importance of adopting a total cardiovascular risk approach to CVD prevention, in alignment with global health goals.

## Introduction

Cardiovascular disease (CVD) is the leading cause of death worldwide.^1^^,^^2^ In low-income and middle-income countries (LMICs), the prevalence of CVD is on the rise, presenting significant public health challenges.^3^^,^^4^ This situation is driven by many factors including inadequate CVD risk factors screening and limited access to timely diagnostic and therapeutic services.^3^^,^^5^ Every year, more than 15 million premature deaths from non-communicable disease (NCD) occur, with around 85% of these in LMICs.^6^ The UN Sustainable Development Goal (SDG) 3.4 urged member states to reduce premature mortality from NCDs by one-third by 2030 through prevention and medical care.^7^

Despite the current high mortality of CVD, combined preventive measures could reduce over half of all CVD events.^8^, ^9^, ^10^, ^11^ A crucial first step is to detect CVD risk factors early to initiate effective prevention.^12^ The single CVD risk approach targets at individual risk factor like high blood pressure or abnormal cholesterol. However, recent evidence shows the total CVD risk depends on age and sex, and the combined effects of risk factors such as high blood pressure, abnormal cholesterol, and smoking.^13^ The WHO recommends the total CVD risk approach for primary prevention in LMICs, which is more cost-effective than the single risk approach, especially in resource-limited settings.^13^^,^^14^

In Myanmar, stroke and ischaemic heart disease were the leading causes of death in 2019.^15^ The WHO STEPwise approach for NCD risk factor surveillance (STEPS) is the standardised method for collecting and analysing national data on key NCD risk factors. According to the SETPS survey, 26.1% of Myanmar’s population were reported to be current tobacco users, 22.4% were overweight, 26.4% had high blood pressure, 10.5% had elevated fasting blood glucose, and 36.7% had high total cholesterol.^16^ In spite of the growing burden of CVD and risk factors in Myanmar, there is no national CVD screening or prevention programme. Myanmar has piloted the WHO Package of Essential NCD interventions (WHO PEN) for early diagnosis of NCDs and risk factors with affordable technologies and medications. However, the WHO PEN pilot has not yet been scaled up across the country. In addition, the frequent stockouts of essential medicines have forced patients to pay out-of-pocket (OOP) or forego treatment.^13^^,^^17^

A National Strategic Plan was issued in 2017 to guide the prevention and control of NCD in Myanmar. However, short of funding and a lack of focus on prevention remain critical challenges to control the NCD in Myanmar.^18^^,^^19^ In 2018, over 90% of CVD expenses were paid by OOP, which is higher than the average 76% OOP for other health services.^20^ In same year, only 8.5% of total CVD expenses were paid by government. In terms of spending, most CVD expenses were used for treatment and only 0.05% of the total CVD expenditure was used for prevention.^20^ Financial barriers created by OOP payments and poor awareness of CVD prevention services has led to under-use of such services.^19^

Given the high prevalence of CVD and its associated risk factors,^16^^,^^21^ and high OOP costs, it is essential for Myanmar to prioritise the implementation of a national CVD prevention strategy to reduce the burden of CVD. To inform such strategy, we conducted a cost-effectiveness analysis (CEA) and budget impact analysis (BIA) on the screening, primary prevention, and secondary prevention of CVDs in Myanmar. The CEA aimed to identify the most cost-effective intervention strategy for CVD prevention in Myanmar. The BIA was framed from a healthcare provider perspective and intended to assess the affordability of different intervention strategies and to provide evidence to inform policymakers for health care budget planning in Myanmar.

## Methods

### Cost-effectiveness analysis

We developed a Markov model in R (version 4.2.2) and Microsoft Excel (v16.0). We conducted a CEA to assess the cost-effectiveness of various CVD prevention strategies from two perspectives. The healthcare provider perspective considered direct medical costs borne by the healthcare provider managed by Ministry of Health and Sports (MOHS). Given premature death and disability are major consequences of CVD, we also applied the societal perspective that considers indirect costs (e.g., productivity costs) in addition to medical costs. We calculated costs and quality-adjusted life years (QALYs) for men and women separately, reflecting their different CVD risk profiles. We then applied the proportion of the male and female population from Myanmar’s census to aggregate results and obtain national level estimates. We compared the different scenarios, measured by the incremental cost-effectiveness ratio (ICER) and net monetary benefit (NMB) (Supplementary Appendix [SA] S1).^22^^,^^23^ We conducted subgroup analysis for different sex and age groups.

### Model structure

**Fig. 1:**
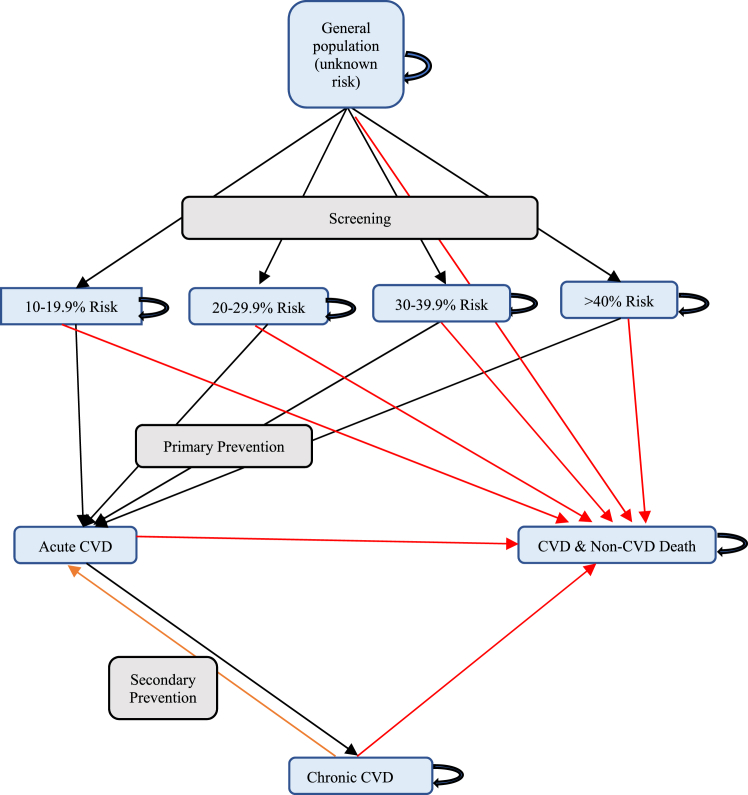
Cardiovascular disease screening and prevention Markov Model. CVD-cardiovascular diseases. Black lines: moving from one health state to another, red lines: moving from any health state to death, orange line: reverse movement from chronic CVD to recurrent acute CVD.

This was a closed cohort study and the model consisted of nine health states: (i) general population with unknown risk; (ii) 10–19.9% risk of having acute CVD (ACVD) within the next 10 years; (iii) 20–29.9% ACVD risk within the next 10 years; (iv) 30–39.9% ACVD risk within the next 10 years; (v) ≥ 40% risk of having ACVD within the next 10 years; (vi) ACVD; (vii) chronic CVD; (viii) CVD-related death; and (ix) non-CVD death (Fig. 1). We described the transitions between these health states in the Markov model (Box 1).

Box 1 **Detailed description of Markov model structure.**

• At the start of the cohort model, individuals aged 45 years with unknown risk in the community were screened.
• Depending on their risk profile, individuals are assigned to 10–19.9% risk or 20–29.9% risk or 30–39.9% risk or ≥ 40% risk groups or remain in the general population (<10% risk).
• Individuals in different risk categories (10–19.9% risk, 20–29.9% risk, 30–39.9% risk or ≥ 40% risk) can stay in the same group or move to ACVD state.
• From the ACVD state, individuals can either go to a CVD-related death or chronic CVD.
• From chronic CVD, individuals can remain in the same health state or move to recurrent ACVD.
• From recurrent ACVD, individuals can either go to an ACVD death or go back to chronic CVD.
• From all health states, some individuals might move to a non-CVD death state.
• Individuals in the model do not move from one risk profile to another as it is difficult to find data on such movement.

CVD is a general term for a group of disorders of the heart and blood vessels; ACVD refers to individuals with acute coronary heart disease and acute stroke; chronic CVD describes individuals with established CVD.

The transition between each health state is specified over a one-year cycle and individuals were assumed to remain in their health state for the entire year. The model is followed over a fifty-five-year period, which is long enough to detect consequences of chronic CVD and simulate the full lifespan of a given individual.

### Scenarios

In alignment with WHO recommendations on CVD preventivion including screening, primary prevention, and secondary prevention,^8^^,^^13^^,^^24^^,^^25^ we developed the following scenarios starting at 45 years of age from the healthcare provider perspective and the societal perspective:

i) No CVD screening or prevention programme (base scenario)
ii) Screening, primary prevention, and secondary prevention of CVD (Sc-PP-SP)
iii) Screening and primary prevention of CVD (Sc-PP)
iv) Secondary prevention of CVD (SP).

#### Base scenario

Some individuals with a high CVD risk would take preventive medication based on the onset of symptoms. Given the absence of national CVD screening or prevention programmes in Myanmar, we used no intervention as a base scenario. Neither the costs nor the health benefits of CVD preventive measures were applied for this scenario.

#### Screening and primary prevention

With our given simulation, the general population with unknown risk and individuals with 10–19.9% risk would visit health centres once a year to screen for CVD risk. Individuals with 20–29.9% risk would receive an angiotensin-converting enzyme (ACE) inhibitor, a calcium channel blocker (CCB), and a statin, go to a health centre twice a year and take laboratory tests annually. Individuals with 30–39.9% risk and ≥40% risk would receive an ACE inhibitor, a CCB, a statin, and aspirin, go to the health centre twice a year, and take laboratory tests annually.

#### Secondary prevention

Individuals with established CVD would receive an ACE inhibitor, a beta-blocker, a statin, and aspirin to prevent recurrent ACVD. They would go to the health centre twice a year and take laboratory tests annually.

#### ACVD treatment

Individuals with ACVD in both the intervention scenarios and base scenario would receive acute treatment. Available treatment options for ACVD in Myanmar are explained in Supplementary Appendix S2.

### Input parameters

The main model parameters and their distributions are shown in Table 1, Table 2. A summary of all the input parameters and sources of information can be found in Supplementary Appendix S3.

**Table 1:** Transition probabilities, mortality rate, and case fatality rate.

Parameters	Values		PSA distribution	References
**TP of risk categories**	**Men**	**Women**		
TP of 10–19.9% risk				
45–54 years	0.25%	0.19%	Beta	WHO “Prevention of cardiovascular disease”: for South-East Asia sub-region D which includes Myanmar^13^
55–64 years	1.12%	1.52%	Beta	
≥65 years	1.81%	0.34%	Beta	
TP of 20–29.9%				
45–54 years	0.08%	0.12%	Beta	
55–64 years	0.57%	0.49%	Beta	
≥65 years	0.01%	0.89%	Beta	
TP of 30–39.9%				
45–54 years	0.06%	0.01%	Beta	
55–64 years	0.35%	0.11%	Beta	
≥65 years	0.49%	0.55%	Beta	
TP of ≥40%				
45–54 years	0.07%	0.05%	Beta	
55–64 years	0.48%	0.47%	Beta	
≥65 years	0.37%	0.33%	Beta	
**TP of CVD**				
TP of CV from 10 to 19.9% risk	1.61%		Beta	WHO “Prevention of cardiovascular disease”: for South-East Asia sub-region D which includes Myanmar^13^
TP of CV from 20 to 29.9% risk	2.84%		Beta	
TP of CV from 30 to 39.9% risk	4.22%		Beta	
TP of CV from ≥ 40% risk	5.80%		Beta	
**TP of recurrent ACVD from chronic CVD**				
45–54 years	6.61%		Beta	Govender et al.: for Middle-Eastern adults^26^
55–64 years	6.84%		Beta	
≥65 years	10.88%		Beta	
**Background mortality rate**				
45–49 years	0.72%	0.40%	Beta	Global health observatory data repository: for Myanmar^27^
50–54 years	1.13%	0.60%	Beta	
55–59 years	1.69%	0.87%	Beta	
60–64 years	2.61%	1.39%	Beta	
65–69 years	3.83%	2.21%	Beta	
70–74 years	5.82%	6.17%	Beta	
75–79 years	8.61%	6.17%	Beta	
80–84 years	13.13%	10.21%	Beta	
≥85 years	21.73%	18.30%	Beta	
**Case fatality rate**				
Acute stroke				WHO OneHealth Tool case fatality rate: for South-East Asia region^28^
40–49 years	26.30%	23.22%	Beta	
50–59 years	21.54%	23.37%	Beta	
60–69 years	21.61%	24.40%	Beta	
70–79 years	27.66%	29.40%	Beta	
≥80 years	38.11%	37.56%	Beta	
Acute ischaemic heart disease				
40–49 years	60.84%	70.94%	Beta	
50–59 years	61.28%	71.30%	Beta	
60–69 years	63.77%	73.07%	Beta	
70–79 years	66.96%	75.18%	Beta	
≥80 years	71.07%	78.50%	Beta	

TP, transition probabilities; CVD, cardiovascular diseases; PSA, probabilistic sensitivity analysis; WHO, World Health Organization.

**Table 2:** Cost, utilities, treatment effects and discount rate.

Parameters	Mean value	Standard error	Distribution	Notes	Reference
Costs					
Cost for screening	9.13	100%	Gamma	Calculated based on services included for each intervention	
Cost for primary prevention (25% risk)	38.62	100%	Gamma		
Cost for primary prevention (>35% risk)	41.11	100%	Gamma		
Cost for secondary prevention	41.28	100%	Gamma		
Cost for ACVD	449.40	100%	Gamma	Calculated by using ratio of ACS and stroke and services provided	
Productivity loss	4360.00	100%	Gamma	Average salary in Myanmar^29^	
**Utilities**					
Utility score for general population	0.93	10%	Beta		Mittmann et al.: for Canadian population^30^
Utility score for having 10–19.9% risk of CVD	0.92	10%	Beta	Estimate	
Utility score for having 20–29.9% risk of CVD	0.91	10%	Beta	Assumed hypertension	
Utility score for having 30–39.9% risk of CVD	0.87	10%	Beta	Assumed diabetes	
Utility score for having ≥ 40% risk of CVD	0.85	10%	Beta	Estimate	
Utility score for having ACVD	0.47	10%	Beta	Calculated by using ratio of ACS and stroke	Matza et al.: for the UK population^31^
Utility score for having chronic CVD	0.64	10%	Beta		
**Treatment effects for primary prevention**					
Relative risk reduction of ACEI, CCB and Statin	0.47	10%	LogNormal		Meta-analyses: for international population^8^^,^^9^^,^^32^, ^33^, ^34^, ^35^
Relative risk reduction of ACEI, CCB, Statin and Aspirin	0.36	10%	LogNormal		
**Treatment effects for secondary prevention**					
Relative risk reduction of ACEI, Beta blocker, Statin and Aspirin	0.29	10%	LogNormal		
Discount rate for costs and outcomes	0.03		Point estimate		

ACEI, angiotensin converting enzyme inhibitor; CCB, calcium channel blocker; CVD, cardiovascular disease; ACVD, acute cardiovascular disease.

#### Transition probabilities (TPs)

##### TPs of different CVD risk and CVD risk scores

The WHO has estimated total CVD risk <10%, 10–19.9% risk, 20–29.9% risk, 30–39.9% risk and >40% risk by different age groups and sex for the southeast Asia region based on risk factors such as blood pressure, cholesterol level, and smoking status (Supplementary Table S1). The incidence of individuals who were in different risk categories was not available for Myanmar. Following the study by Schaufler and Wolff,^36^ the annual TP of 10–19.9% risk, 20–29.9% risk, 30–39.9% risk, and ≥40% risk was calculated from WHO age- and sex-dependent prevalence data, assuming constant prevalence over time (Supplementary Appendix S3 and Table S1).

##### TPs of ACVD from different CVD risk

The total risk profile gives a numerical probability of ACVD occurring in the next ten years. Individuals in the 10–19.9% risk category have a 10–19.9% risk of developing CVD over the next 10 years. For TP calculation, we used the average value of each risk group, e.g., 15% for the 10–19.9% risk group. This 10-year incidence was converted into an annual TP.^37^

##### TPs of recurrent ACVD from chronic CVD

The TP from patients with a history of CVD (chronic CVD) to recurrent ACVD was taken from a retrospective cohort study conducted by Govender and colleagues^26^ at a large tertiary hospital in the United Arab Emirates.

#### Costs

Myanmar's health care system is a mixed private and public system.^38^ Prices of health services vary greatly among private sector providers. Public sector providers are supposed to provide free health care to the public. We estimated the costs from a public healthcare provider perspective. We referred to WHO guidelines and defined a standard package for each health state.^8^^,^^13^^,^^24^^,^^25^ For each scenario, we identified drugs, tests, clinical services, and other medical interventions needed and estimated the total costs by multiplying unit price with quantity (Supplementary Appendix S4 and Tables S5–S7).

We collected medication prices from the International Medical Products Price Guide for 2015 published by Management Science for Health in collaboration with the WHO.^39^ In this study, we used the buyer price for medications, which is the government agency international competitive bidding (or tender) price.^39^ We chose the lowest price because healthcare providers in LMICs are encouraged to use generic medicines. We obtained the cost for outpatient health centre visits and hospital beds per day from WHO “CHOsing Interventions that are Cost-Effective” (WHO-CHOICE) estimates for Myanmar.^40^ We collected the costs of laboratory tests and treatments from private laboratories and hospitals and then converted these into an equivalent public hospital cost. The average salary in Myanmar was used as productivity loss from a societal perspective.^41^ Detailed costs are shown in SA4.

#### Utilities

We used QALYs, a standardised measure of disease burden that combines the quantity and quality of life into a single index, to reflect health outcomes. The utility of the general population was assigned with the utility score of people without a chronic condition.^30^ As individuals’ CVD risk was predicted based on CVD risk factors like hypertension and diabetes, they are likely to have either hypertension or diabetes or both.^13^ Hence, we assumed that individuals with 20–29.9% CVD risk are equivalent to the state of hypertension and those with 30–39.9% CVD risk are equivalent to the state of diabetes. We then assumed that individuals with 10–19.9% CVD risk would have higher health utility than those with 20–29.9% CVD risk and people with ≥40% CVD risk would have lower health utility than those with 30–39.9% CVD risk. We used the health utilities for acute and chronic CVD from a study on the United Kingdom population.^31^

#### Treatment effect

The effectiveness of primary prevention and secondary prevention were derived from meta-analysis data (Supplementary Table S2)^8^^,^^9^^,^^32^, ^33^, ^34^, ^35^ As treatment effects were estimated separately for ischaemic heart disease and stroke, the weight of the prevalence of these two diseases was used to create a composite CVD treatment effect. The effects for the medications were assumed to be independent, and therefore the overall effect was calculated by multiplying the individual relative risk associated with each medication.^8^^,^^11^^,^^37^

### Probabilistic sensitivity analysis (PSA)

We used Monte Carlo simulation to conduct PSA. The model parameters were assumed to vary according to specific distributions.^37^ We used gamma distributions for cost parameters, beta distributions for utilities, and transition probabilities and lognormal distributions for the treatment effects.

### Budget impact analysis

We examined the annual impact on budget and health outcomes for adopting the three previously mentioned intervention scenarios (Sc-PP-SP, Sc-PP and SP, respectively), compared to the base scenario from the healthcare provider perspective over 3 years.^42^^,^^43^ A detailed description of BIA can be found in Supplementary Appendix S1.

### Role of the funding source

The funders had no role in study design, data collection, data analysis, interpretation, or writing of the report.

## Results

### Cost-effectiveness analysis

From a healthcare provider perspective, SP is the most cost-effective option at the threshold of one time the GDP per capita. However, the Sc-PP-SP intervention became the most cost-effective option at the threshold of three times the GDP per capita. From a societal perspective, the Sc-PP-SP intervention is the most cost-effective strategy at both thresholds (Table 3).

**Table 3:** Cost-effectiveness analysis: costs, benefits, incremental cost-effectiveness ratio, and net monetary benefits.

Interventions	Costs per person (US$)	Effects per person (QALYs)	ICERs (Cost/QALY)		NMB (one time GDP-US$ 1400.2)	NMB (three times GDP-US$ 4200.6)
From healthcare provider perspective						
No screening or prevention	34.66	17.60			24,609.67	73,898.32
Secondary prevention only	39.40	17.63	160.42	160.42	**24,646.32**	74,017.77
Screening, primary prevention only	235.52	17.74	1821.68 (E/D)		24,600.94	74,273.88
Screening, primary & secondary prevention	238.55	17.76	160.10	1573.74	24,624.36	**74,350.17**
**From societal perspective**						
Secondary prevention only	348.56	17.63			24,337.16	73,708.61
No screening or prevention	361.92	17.60	S/D		24,282.40	73,571.05
Screening, primary & secondary prevention	430.46	17.76	647.17	647.17	**24,432.45**	**74,158.26**
Screening, primary prevention only	438.60	17.74	S/D		24,397.86	74,070.80

ICER, incremental cost-effectiveness ration; E/D, extended dominance; GDP, gross domestic products; NMB, net monetary benefits; QALY, quality adjusted life years; S/D, strong dominance; US$, United States Dollar.

The most cost-effective strategy, indicated by the highest net monetary benefit values (NMB) at given threshold levels is bolded.

Subgroup analysis shows that intervention starting at age 45 years is the most cost-effective option for men and women, across all scenarios and from both perspectives. Notably, interventions showed greater cost-effectiveness among women compared to men across all strategies (Table 4).

**Table 4:** Cost-effectiveness analysis: costs, benefits, incremental cost-effectiveness ratio, and net monetary benefits for subgroup analysis.

Interventions	Costs per person (US$)	Effects per person (QALYs)	ICERs (Cost/QALY)		NMB (GDP per capita-US$ 1400.2)	NMB (three times GDP per capita-US$ 4200.6)
Healthcare provider perspective						
Men						
*Screening, primary & secondary prevention*						
Starting at 65 years	126.99	9.634			13,361.85	40,339.53
Starting at 55 years	197.20	13.095	20.28 (E/D)		18,138.06	54,808.57
Starting at 45 years	221.41	16.713	6.69	13.34	**23,179.95**	**69,982.67**
*Screening and primary prevention only*						
Starting at 65 years	125.97	9.627			13,354.31	40,314.88
Starting at 55 years	194.44	13.078	19.84 (E/D)		18,117.28	54,740.71
Starting at 45 years	218.66	16.697	6.69	13.11	**23,160.23**	**69,918.01**
*Secondary prevention only*						
Starting at 65 years	22.30	9.586			13,400.66	40,246.59
Starting at 45 years	36.51	16.605	2.02	2.02	**23,213.41**	**69,713.24**
Starting at 55 years	43.10	12.969	SD		18,115.49	54,432.67
Women						
*Screening, primary & secondary prevention*						
Starting at 65 years	171.32	11.301			15,652.58	47,300.38
Starting at 55 years	232.49	15.048	16.33 (E/D)		20,837.81	62,978.43
Starting at 45 years	252.56	18.611	5.63	11.12	**25,806.15**	**77,923.58**
*Screening and primary prevention only*						
Starting at 65 years	170.30	11.295			15,644.35	47,273.63
Starting at 55 years	229.04	15.025	15.75 (E/D)		20,808.88	62,884.72
Starting at 45 years	249.32	18.590	5.69	10.83	**25,779.71**	**77,837.77**
*Secondary prevention only*						
Starting at 65 years	29.24	11.186			15,632.94	46,957.29
Starting at 45 years	41.77	18.469	1.72	1.72	**25,818.70**	**77,539.66**
Starting at 55 years	50.25	14.880	S/D		20,784.94	62,455.31
**From societal perspective**						
Men						
*Screening, primary & secondary prevention*						
Starting at 65 years	126.99	9.634			13,361.85	40,339.53
Starting at 55 years	317.64	13.095	55.08 (E/D)		18,017.62	54,688.13
Starting at 45 years	409.40	16.713	25.36	39.89	**22,991.96**	**69,794.68**
*Screening and primary prevention only*						
Starting at 65 years	125.97	9.627			13,354.31	40,314.88
Starting at 55 years	318.93	13.078	55.92 (E/D)		17,992.79	54,616.23
Starting at 45 years	418.68	16.697	27.56	41.40	**22,960.21**	**69,717.99**
*Secondary prevention only*						
Starting at 65 years	22.30	9.586			13,400.66	40,246.59
Starting at 55 years	259.09	12.969	70.01 (E/D)		17,899.50	54,216.68
Starting at 45 years	350.21	16.605	25.06	46.72	**22,899.70**	**69,399.53**
Women						
*Screening, primary & secondary prevention*						
Starting at 65 years	171.32	11.301			15,652.58	47,300.38
Starting at 55 years	371.56	15.048	53.44 (E/D)		20,698.75	62,839.37
Starting at 45 years	447.68	18.611	21.37	37.81	**25,611.03**	**77,728.47**
*Screening and primary prevention only*						
Starting at 65 years	170.30	11.295			15,644.35	47,273.63
Starting at 55 years	372.22	15.025	54.13 (E/D)		20,665.71	62,741.55
Starting at 45 years	454.91	18.590	23.20	39.01	**25,574.13**	**77,632.19**
*Secondary prevention only*						
Starting at 65 years	29.24	11.186			15,632.94	46,957.29
Starting at 55 years	270.95	14.880	65.43 (E/D)		20,564.23	62,234.60
Starting at 45 years	347.22	18.469	21.25	43.66	**25,513.26**	**77,234.21**

ICER, incremental cost-effectiveness ration; E/D, extended dominance; GDP, gross domestic product; NMB, net monetary benefits; QALY, quality adjusted life years; S/D, strong dominance; US$, United States Dollar.

The most cost-effective strategy, indicated by the highest net monetary benefit values (NMB) at given threshold levels is bolded.

### Probabilistic sensitivity analysis

We presented the cost-effectiveness acceptability curves (CEACs) based on 10,000 Monte Carlo simulations to estimate the probability of cost-effectiveness at a chosen threshold level (Supplementary Appendix S5). CEACs were plotted from a range of cost-effectiveness thresholds (willingness to pay) on the x-axis against the probability of cost-effectiveness on the y-axis.

**Fig. 2:**
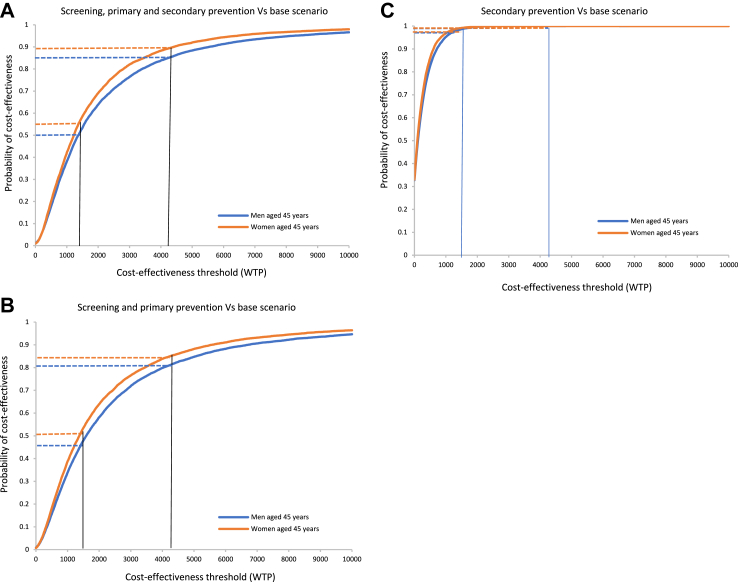
Cost-effectiveness acceptability curves from a healthcare provider perspective showing a probability of cost-effectiveness for one-to three-time Myanmar’s GDP per capita.

For the scenario of Sc-PP-SP, there was a 50.86% and 55.84% probability of cost-effectiveness for men and women, respectively, at the one time the GDP per capita threshold and 84.82% and 89.18%, respectively, at the three times the GDP per capita threshold. Meanwhile, the probability of cost-effectiveness for the Sc-PP scenario was 45.34% (men) and 50.74% (women) at the one time the GDP per capita threshold and 80.87% (men) and 84.79% (women) at the three times the GDP per capita threshold. The SP strategy had a high probability (>98%) of being cost-effective for men and women even at the one time the GDP per capita threshold (Fig. 2).

The CEACs for Sc-PP-SP, Sc-PP and SP from a societal perspective and the cost-effectiveness planes for Sc-PP-SP are shown in Supplementary Appendix S5.

### Budget impact analysis results from a healthcare provider perspective

For the Sc-PP-SP strategy, we estimated to provide screening intervention to 11 million individuals. Of these, about 2 million individuals would receive primary prevention, and 31,000 people would be eligible for secondary prevention in Year 1 of CVD preventive care. These services would prevent 55,000 ACVD events and avert 28,000 CVD-related deaths. It would cost the health system around US$ 182 million (screening: US$ 100 million; primary prevention: US$ 81 million; secondary prevention: US$ 1.3 million). With 25 million in cost savings from ACVD treatment, the total budget needs would be US$ 157 million (Table 5). For a 50% coverage of Sc-PP-SP, the total budget would be USD 79 million, resulting in 28,000 ACVD events prevented, and 14,000 CVD-related deaths averted. At 75% coverage, the total budget would be US$ 118 million with 42,000 ACVD events prevented, and 21,000 CVD-related deaths averted (Supplementary Appendix S5 and Table S8).

**Table 5:** Budeget impact analysis: eligible population, budget impacts, and health benefits for Year 1.

	Year 1		
	Sc-PP-SP	Sc-PP	SP
**Eligible population**			
For screening	10,948,870	10,948,870	
For primary prevention (25%)	820,106	820,106	
For primary prevention ( ≥ 35%)	1,184,545	1,184,545	
For secondary prevention	31,306		57,152
**Budget**			
Total intervention related cost	**182,136,979**	**180,836,771**	**2,373,641**
i) Screening	99,994,487	99,994,487	
ii) Primary prevention	80,842,284	80,842,284	
iii) Secondary prevention	1,300,208		2,373,641
**Cost saving from reduced ACVD for specific intervention(s)**			
ACVD treatment cost without intervention	53,581,979	51,444,681	2,137,298
ACVD treatment cost with intervention	28,695,628	28,352,329	628,621
Changes in disease related cost	**24,886,350**	**23,092,351**	**1,508,677**
**Budget impacts**	**157,250,628**	**157,744,420**	**864,964**
**Health benefits**			
Reduced number of ACVD	55,377	51,385	3357
Reduced number of CVD death	27,559	25,539	1699

ACVD, acute cardiovascular disease; CVD, cardiovascular disease; PP, primary prevention; Sc, screening; SP, secondary prevention.

For the Sc-PP strategy, the Year 1 budget would be US$ 158 million. Despite incurring higher cost than the Sc-PP-SP strategy due to reduced cost saving from ACVD treatment, this strategy would achieve fewer health benefits than the Sc-PP-SP strategy. Specifically, it would prevent 51,000 ACVD events and avert 26,000 CVD-related deaths (Table 5).

The budget impact for the SP strategy would be US$ 864,000 in Year 1. However, it would have the least health benefits among the three strategies. The SP strategy would reduce 3357 ACVD events and save 1699 ACVD deaths (Table 5).

The BIA results for Years 2 and 3 and total of all three years of preventive CVD care (Years 1–3) are shown in Supplementary Tables S9 nand S10. Over three years, the budget impact for Sc-PP-SP would be US$ 441 million with 159,000 ACVD prevented, and 80,000 CVD-related deaths averted. Over the same period, the Sc-PP strategy would cost US$ 443 million, preventing 137,000 ACVD events and averting 69,000 CVD-related deaths. The budget impact for SP would be US$ 5 million with 18,000 CVD events prevented and 9000 CVD-related deaths averted.

## Discussion

We found that Sc-PP-SP is the most cost-effective strategy at the three times the GDP per capita threshold from the healthcare provider perspective. It is also the most cost-effectiveness strategy from the societal perspective at both threshold levels. However, the SP is the best option at the one time the GDP per capita threshold from a healthcare provider perspective. With approximately 11 million individuals qualifying for Sc-PP-SP, the estimated budget to be US$ 157 million in Year 1 with 55,000 ACVD events prevented and 28,000 ACVD deaths averted.

Among different age groups, we found initiating intervention at the age of 45 years is the most cost-effective, possibly due to its higher QALYs compared to interventions starting at a later age. In comparison with men, preventive interventions for women are more cost-effective, potentially due to the higher life expectancy of women in Myanmar.

Previous studies have shown that most CVD screening and prevention interventions are cost-effective for LMICs although intervention strategies in these studies varied. Gaziano and colleagues assessed multidrug regimens for primary and secondary prevention of CVD in six regions. The ICERs of primary prevention ranged from US$ 746–1221/QALY gained while ICERs of secondary prevention were US$ 306–388/QALY gained across different regions compared to no treatment.^8^ Khonputsa and colleagues found that primary CVD prevention with a polypill (three blood pressure-lowering drugs and a statin) or a combination of three generic blood pressure-lowering drugs is cost-effective for the Thai population.^44^ Nguyen and colleagues observed screening and managing identified hypertension for CVD prevention in Vietnam were generally cost-effective though the cost per QALY gained varied across interventions and age groups.^45^ Megiddo and colleagues reported that secondary prevention with aspirin and beta-blockers at 80% coverage would be highly cost-effective and the addition of ACE inhibitors would also be cost-effective in India.^46^ Another CEA study in India conducted by Basu and colleagues found that all primary and secondary prevention interventions would be cost-effective across a broad range of access and adherence levels.^47^

Our study has several strengths. To our knowledge, this is the first study to report both the cost effectiveness and budget impact of CVD prevention for Myanmar. For a resource-limited country like Myanmar, such evidence could be very useful to inform the resources allocation within the nation’s constrained budget. Our model provides comprehensive information by including various preventive interventions—screening, primary prevention, and secondary prevention. Importantly, this model closely mirrors the natural disease progression, reflecting the “real world” situation.

We believe that our findings could have impacts on Myanmar’s national CVD prevention policy in several significant ways. First, our study provides evidence to support policy makers to scale up the existing WHO PEN pilot, a critical step in tackling NCDs across the country. Myanmar’s WHO PEN project started in two townships in 2012 and expanded to 20 townships in 2017. Between May 2017 and December 2018, the project screened a total of 152,446 individuals for NCD risk factors, which is much lower than the estimated eligible population. Even with such low coverage, shortage of essential equipment and medicines remains a major challenge that is hindering Myanmar’s PEN project.^17^ Evidence of the cost-effectiveness of CVD prevention from this study will hopefully encourage decision makers to allocate more resources to scale up CVD prevention programmes nationwide.

Second, our findings also shed light on the low prioritisation of investment in CVD prevention programmes. At present, NCD control gets less attention, less international aid, and less of the government budget than communicable diseases. Our findings underscore the need to prioritise NCD control efforts and catalyse increased funding from both the government and donors. Such a support will lift the financial barriers and improve access to CVD prevention services.^48^ It will be challenging for the government to fund CVD prevention programmes. However, in the short-term, our evidence on cost-effectiveness can be used to advocate for more financial support from development partners who are currently contributing very little development assistance to NCDs. According to Myanmar’s National Health Accounts, in 2018, international aid contributed 66% of the cost of the ‘HIV/AIDS and other sexually transmitted diseases programmes, 65% of tuberculosis expenses, and 91% of malaria programmes. In the same year, only 0.56% of CVD programmes were funded by international aid.^20^ The report of the WHO Commission on Macroeconomics and Health suggested that the international community should support interventions whose ICER is less than three times the GDP per capita if the country cannot afford these interventions.^49^ At the same time, ministries of health should focus on developing prepayment mechanisms, such as extending existing social health insurance, subsidising the informal workers and population in poverty, and increasing tax revenues by earmarking health taxes on cigarettes, alcohol, and fossil fuels over the long term. Third, the single risk factor approach is still a widespread practice in Myanmar, with exception to the WHO PEN pilot sites. Our study used the total cardiovascular risk factor approach recommended by the WHO, which could potentially inform the clinical practice change.

This study has several limitations. First, some parameters were obtained from different studies as some data are not available in Myanmar. We recognise this could introduce uncertainty to the CEA results. However, the probabilistic sensitivity analysis and cost-effectiveness acceptability curves have been used to illustrate the robustness of our findings under different conditions. Second, the model did not include transitions from a high-risk category to a low-risk category for individuals on preventive medicines. Third, regarding lifelong medications needed for CVD prevention, we did not consider the adherence rate to treatment. Fourth, we used average unit cost for all parameters, and we did not consider economies or diseconomies of scale in our budget impact analysis. Fifth, for costs, we used the conservative approach of visiting a health centre and laboratory tests for screening and prevention of CVD risk. The WHO PEN pilot in Myanmar encourages the use of low technology and inexpensive investigations such as glucometers, glucostrips and lipid analysers, and local basic health staff for NCD prevention programme. Our budget estimates would have been significantly lower if we had used services provided by the WHO PEN project. Sixth, the cost of treatment for medication side effects was not included. However, these medications have been widely used globally with very few side effects.

Despite the limitations, this study has significant implications for Myanmar's policymakers by informing the decisions on national health programme and budget planning. The CEA findings will support the decision makers in Myanmar to invest in proven, cost-effective programs and to use scarce healthcare resources effectively and efficiently. The BIA results, including the eligible population for intervention, the total budget needs, and health benefits, could be helpful tool for effective budget planning for the CVD prevention programmes nationwide. As stroke and ischaemic heart disease persist as leading causes of death in Myanmar, the implementation of a comprehensive prevention programme national wide, such as the WHO PEN project, could substantially reduce CVD-related deaths and improve quality of life. These endeavours align with the pursuit of Sustainable Development Goals (SDG) 3.4, which aims to reduce premature mortality from NCDs by one-third by the year 2030 through prevention and treatment.

## Contributors

WM, ZMW, TT, ZLK, PYP, OO and GY conceptualised this study. ZMW led the data collection and analysis with inputs from WM, TT, ZLK and PYP. ZMW drafted the manuscript with contributions from all co-authors. WM, TT, OO, and GY edited and revised the final manuscript and all authors read and approved the final version.

## Data sharing statement

All the data we used is publicly available and sources of information can be found in Methods and Supplementary Appendix.

## Declaration of interests

This project was funded by a pilot grant from Duke Global Health Institute, USA. WM, OO and GY have received grants from WHO, and Bill and Melinda Gates Foundation (payments made to Centre for Policy Impact in Global Health, Duke University); ZM, TT, ZLK, and PYP have received grants from Bill and Melinda Gates Foundation (payments made to Community Partners International through Centre for Policy Impact in Global Health, Duke University). Authors declare no other conflicts of interest.
