# Supplementary material for: Cost-effectiveness and budget impact analysis of screening and preventive interventions for cardiovascular disease in Myanmar: an economic modelling study
Source: Lancet Reg Health Southeast Asia. 2024 Apr 10;26:100394. doi: 10.1016/j.lansea.2024.100394 (PMC11022086; doi:10.1016/j.lansea.2024.100394)
Supplement: Supplementary Appendix S1–S5 [file mmc1.docx]

**Supplementary information**

Table of Contents

[Supplementary Appendix 1. Analysis: Incremental cost-effectiveness ration (ICER), net monetary benefits (NMB) and budget impact analysis (BIA) 2](#_Toc140594612)

[ICER and NMB 2](#_Toc140594613)

[BIA 2](#_Toc140594614)

[Supplementary Appendix 2. Detailed services and available treatment options for acute cardiovascular disease (ACVD) 3](#_Toc140594615)

[Supplementary Appendix 3. Input parameters 4](#_Toc140594616)

[Supplementary Appendix 4. Costs 8](#_Toc140594617)

[Supplementary Appendix 5. Results: probabilistic sensitivity analysis (PSA) and budget impact analysis (BIA) 11](#_Toc140594618)

[PSA results 11](#_Toc140594619)

[BIA results 14](#_Toc140594620)

[References: 17](#_Toc140594621)

# Supplementary Appendix 1. Analysis: Incremental cost-effectiveness ration (ICER), net monetary benefits (NMB) and budget impact analysis (BIA)

## ICER and NMB

Cost effectiveness analysis was performed by calculating incremental cost effectiveness ratio (ICER) and net monetary benefit (NMB). For ICER calculation, all strategies were ranked in ascending order by cost, with the lowest cost at the top. First, the strongly dominated strategies—higher costs with less benefits—were identified and ruled out. Then, ICERs were calculated between adjacent pairs of non-dominated strategies, and extendedly dominated strategies—a higher ICER than the strategy immediately below it—were ruled out. After that, ICERs were recalculated between remaining adjacent pairs of non-dominated strategies. Finally, ICERs results were compared with the threshold level to identify the most cost-effective option. While ICER calculation needs pairwise comparison, NMB calculation does not. The NMB for each strategy was calculated by multiplying the quality adjusted life years (QALYs) with the chosen threshold level and then subtracting the cost of each strategy. The strategy with the highest NMB value is considered the most cost-effective one, while the strategy with the lowest NMB is the least cost-effective one. Although calculations are different, these two approaches give the same conclusion about the most cost effective strategy for any given threshold level.^1,2^

## BIA

In this BIA, Myanmar 2014 census data, the proportion of the population in each CVD risk category for the South-East Asia region, including Myanmar, and the prevalence of CVD in Myanmar were used to calculate the eligible population: the number of individuals who were in each risk category (<10%, 10–19.9%, 20–29.9%, 30–39.9%, and ≥40%).^3–5^ An open cohort with the same annual transition probabilities in the Markov model was used to move individuals from risk groups to ACVD, then to ACVD death or chronic CVD. From chronic CVD, some patients will move to recurrent ACVD and then to CVD death, with the remaining patients moving back to chronic CVD (Table S1-S8).

In the BIA, in the first year, all 45 years old and above without established CVD were provided screening. Individuals with established CVD were excluded by using prevalence of heart attack and stroke in Myanmar.^4^ Individuals with 20–29·9% risk, 30–39·9% risk, and ≥40% risk of having an ACVD event were given primary preventive medicines. When individuals received primary prevention treatment, we used the treatment effect of medication so that fewer individuals would have ACVD, ACVD death, and related costs compared to the no-intervention program.

Individuals who survived ACVD were provided secondary preventive medicines. When individuals received secondary prevention treatment, we used the treatment effect of medication so that fewer individuals would have recurrent ACVD, recurrent ACVD death, and its related costs compared to the no-intervention program.

Individuals who did not have an ACVD event will be carried forward to their corresponding risk groups for subsequent years. A new population eligible for screening (individuals who become 45 years old but did not have established CVD) was added, and individuals who died were subtracted using age-specific background mortality rate. New individuals and those with a <10% risk and 10–19·9% risk received yearly screening. Individuals with 20–29·9% risk, 30–39·9% risk, and ≥40% risk continue to receive primary prevention and move to health states like those in the first year. Individuals in chronic CVD and those who survived from recurrent CVD will be carried forward to subsequent years. Individuals who died from background mortality were subtracted. They would continue to receive secondary prevention and move to health states like those in the first year. Costs for each health state (Table 2) were applied to corresponding eligible population.

# Supplementary Appendix 2. Detailed services and available treatment options for acute cardiovascular disease (ACVD)

**ACVD treatment:** Individuals with ACVD in both the intervention scenarios and base scenario would receive acute treatment. For acute coronary syndrome, ST-segment elevation myocardial infarction (STEMI) will require percutaneous coronary intervention (PCI). However, PCI is available only in urban settings. We estimated the percentage of patients with acute coronary syndrome (ACS) who would receive PCI by multiplying the proportion of patients with ACS who have STEMI with the proportion of Myanmar’s population that live in urban settings (estimated 60·6% STEMI *30% urban population = 18·18%) as detailed in previous studies for low-income and middle-income countries.^5–7^ The rest of the patients with STEMI (42·42% of those with STEMI) would receive medical treatment with thrombolysis. Non-STEMI patients (39·4%) would receive medical treatment without thrombolysis. For stroke, there is no resource and expertise for medical thrombectomy in Myanmar, and 5 government and 5 private hospitals have offered only a few hundred thrombolysis treatments since 2015.^8,9^ Hence, we assume most of the patients with stroke are provided with medical supportive care.

# Supplementary Appendix 3. Input parameters

**Table S1. Proportion of population in each CVD risk category for southeast Asia region including Myanmar**

|  | **Men** |  |  |  | **Women** |  |  |  |
| --- | --- | --- | --- | --- | --- | --- | --- | --- |
| Risk Category | <50 years | 50-59 years | 60-69 years | 70+ years | <50 years | 50-59 years | 60-69 years | 70+ years |
|  | % | % | % | % | % | % | % | % |
|  |  |  |  |  |  |  |  |  |
| 10-19·9% | 1·3 | 7·45 | 27·48 | 44·02 | 0·65 | 8·72 | 38·52 | 40·66 |
| 20-29·9% | 0·25 | 4·41 | 14·32 | 14·42 | 0·74 | 3·39 | 12·45 | 22·95 |
| 30-39·9% | 0·23 | 2·13 | 9·48 | 15 | 0·09 | 0·28 | 6·97 | 14·25 |
| >=40% | 0·24 | 2·99 | 12·75 | 16·39 | 0·13 | 3·03 | 12·26 | 15·5 |
|  |  |  |  |  |  |  |  |  |

Source: Prevention of cardiovascular disease: guidelines for assessment and management of cardiovascular risk. Geneva: World Health Organization; 2007^3^

As mentioned in Schaufler and Wolff, 2010, incidence rates of different risk categories are calculated from WHO’s age and sex dependent prevalence data by using following formula, assuming constant prevalence over time.^10^

IR=Pt/1-Pt ((P$\bar{t}$/Pt))^1/∆t^-1)

IR-yearly incidence

Pt- prevalence of the lowest (youngest)

P$\bar{t}$ -prevalence of the highest (oldest)

∆t – age difference between the two cohorts

**Table S2. Input parameters: treatment effects for each medicine**

| **Parameters** | **Values** | | **References** |
| --- | --- | --- | --- |
| **Treatment effect** |  |  |  |
| Primary prevention | **IHD** | **Stroke** |  |
| Aspirin | 0·68 | 0·84 | Meta-analyses: for international population^11–16^ |
| Statin | 0·64 | 0·94 |  |
| ACEI and CCB | 0·66 | 0·51 |  |
| Secondary prevention |  |  |  |
| Aspirin | 0·66 | 0·78 |  |
| Statin | 0·71 | 0·81 |  |
| ACEI | 0·8 | 0·68 |  |
| Beta blocker | 0·73 | 0·71 |  |
|  |  |  |  |

ACEI-angiotensin converting enzyme inhibitors, CCB- calcium channel blockers, CVD- cardiovascular diseases, ACS- acute coronary syndromes

**Table S3. A summary of inputs and sources of information**

| **Inputs** | **Subgroups** | **Sources** | **Technical notes** |
| --- | --- | --- | --- |
|  |  |  |  |
| TP of different cardiovascular risk and total risk scores | By age and gender | WHO "Prevention of cardiovascular disease"^3^ | South-East Asia Region D which includes Myanmar |
| TP of acute CVD | By risk scores | WHO "Prevention of cardiovascular disease"^3^ | South-East Asia Region D which includes Myanmar |
| TP of recurrent acute CVD | By age | Govender et al.: Incidence and risk factors for recurrent cardiovascular disease in middle eastern adults^17^ | Retrospective cohort study conducted in a large tertiary hospital providing health care services to UAE nationals and the expatriate community. The study showed Incidence rates of recurrent acute CVD events that can be calculated to TP. |
| Mortality rates | By age and gender | Global health observatory data repository^18^ | Myanmar |
| Case fatality rates | By age and gender | WHO OneHealth Tool case fatality rate^19^ | South-East Asia region |
| Cost for medications | per tablet/ per year | International medical products price guide^20^ | Buyer price, lowest price (2020 US$) |
| Cost per outpatient visit | per visit | WHO-CHOICE^21^ | For health centre (no bed) in Myanmar (2019 US$) |
| Cost per inpatient day | per day | WHO-CHOICE^21^ | For secondary hospital in Myanmar (2019 US$) |
| Cost for laboratory tests | per test/ per service | Than et al.: Unit cost of healthcare services at 200-bed public hospitals in Myanmar; and costs from private laboratories^22^ | Costs from private laboratories were adjusted to laboratory costs from public hospitals. This hospital based cross-sectional study was done at two hospitals in Myanmar. (2021 US$) |
| Investigation cost for acute CVD: Cardiac biomarkers, ECG, Echocardiogram, CT/MRI head and other laboratory tests | per test | From private hospitals | Used adjustment figure |
| Treatment mix for acute coronary syndrome | ST segment elevation myocardial infarction (STEMI) and non-STEMI | Literature for low-income and middle-income countries^5–7^ | STEMI in urban setting are to receive percutaneous coronary intervention. STEMI in rural setting are to receive medical treatment with thrombolysis. Non-STEMI are to receive medical treatment without thrombolysis |
| Percutaneous coronary intervention for MI | per package | From private hospital | Package price for PCI from private hospital was adjusted to an equivalent public hospital. (2020 US$) |
| Treatment mix for stroke | for acute stroke | Literature from Myanmar^8,9^ | No resource and expertise for mechanical thrombectomy. A small number of stroke patients receive thrombolysis. Most of the stroke patients are to receive medical supportive care only. |
| Salary | annual salary | salary explorer^23^ | Average salary in Myanmar (2021 US$) |
| Currency | MMK and US$ | WB world development indicators^24^ | Adjusted to 2020 US$ by using WB CPI for Myanmar currencies and CPI calculator for US$. The last CPI available for Myanmar is 2019, therefore, some price can only be adjusted to the nearest available value |
| Utilities for different risk categories | by health states | Mittmann et al.: Utility scores for chronic conditions in a community-dwelling population^25^ | For Canadian population in community |
| Utilities for acute and chronic CVD | by health states | Matza et al.: Acute and chronic impact of cardiovascular events on health state utilities^26^ | For the UK population |
| Treatment effects | for each medicine/ for each intervention | Meta-analysis^11–16^ | For international population |
| Discount rate | by cost and outcome |  | 3% discount rate |
| Incidence of acute CVD^1^ | by disease (MI and Stroke) | Swe et al.: Increasing trends in admission due to non-communicable diseases over 2012 to 2017: findings from three large cities in Myanmar^27^ | Cross-sectional study using medical records from tertiary hospitals in Myanmar |
| Population and composition^2^ | By age and gender | 2014 Census^5^ | Myanmar population |
|  |  |  |  |

ACS- Acute coronary syndrome, CPI- Consumer price index, CVD- Cardiovascular diseases, MI- Myocardial infarction, MMK- Myanmar Kyat, PCI- Percutaneous coronary intervention, TP- Transition probabilities, UK- United Kingdom, US$- United States Dollar, WB- World Bank, WHO-CHOICE- Choosing interventions that are cost-effective

^1^ Composite acute CVD includes myocardial infarction (MI) and cerebrovascular diseases (stroke). The number of admissions of cardiovascular diseases (17766 stroke [58%] vs. 12687 ischemic heart disease [42%] over 2012-2017) for Myanmar was used to weight treatment effects, utilities, costs, and other composite CVD-related parameters. ^27^

^2^ The total number of the population aged 45 years and over by gender from “The 2014 Myanmar Population and Housing Census”^5^ are shown in Table S3. The 43-year-olds and the 44-year-olds who would become 45-year-olds in the next one and two years are also shown.

**Table S4. Myanmar population by age and sex from Myanmar census 2014**^5^

| **Age** | **Male** | **Female** | **Total** |
| --- | --- | --- | --- |
| 43 | 304,002 | 341,823 | 645,825 |
| 44 | 261,167 | 299,316 | 560,483 |
| 45-49 | 1,375,041 | 1,571,107 | 2,946,148 |
| 50-59 | 2,118,320 | 2,492,849 | 4,611,169 |
| 60-69 | 1,178,658 | 1,462,680 | 2,641,338 |
| 70+ | 742,682 | 1,090,388 | 1,833,070 |
|  |  |  |  |

# Supplementary Appendix 4. Costs

We collected the costs of laboratory tests for fasting glucose, cholesterol, complete blood count, urea and electrolytes, and liver function from two private laboratories and calibrated with estimates of laboratory tests from two public hospitals where the unit cost of health care service was calculated using top-down costing approach.^22^ Costs from the public sector were less than 50% of private hospital costs and we applied an approximate 50% fraction to adjust the costs from private hospitals to public hospitals. The cost of percutaneous coronary intervention for acute myocardial infarction (MI) treatment was obtained from a private hospital and adjusted to an equivalent public hospital cost using the previous adjustment figure. Costs for radiology, imaging tests and extra nursing care, etc. for medical treatment of MI and stroke were estimated using costs from private hospitals and the literature.

All costs were reported or adjusted to the 2020 US$ equivalent or nearest figure possible using the consumer price index.^24,28^ Myanmar’s currency was converted to United States dollars using a currency exchange rate of 1 US$ = 1500 MMK.^29^ Unit costs of all parameters, such as drugs, laboratory tests, and health centre visits are summarised in Table S5, costs per year/service are in Table S6, and costs for each health state are in Table S7.

**Table S5. Input parameters: cost per unit**

| **Parameters** | **per unit (US$)** | **References** |
| --- | --- | --- |
|  |  |  |
| Amlodipine 10 mg | 0·0051 | International medical products price guide^20^ |
| Enalapril 10 mg twice | 0·0063 |  |
| Atorvastatin 20 mg | 0·0200 |  |
| Aspirin 100mg | 0·0062 |  |
| Clopidogrel | 0·0822 |  |
| Atenolol 50 mg | 0·0055 |  |
| Streptokinase per vial | 35·32 |  |
| Fasting Glucose | 1·00 | From private laboratory in Myanmar (adjusted to cost of public laboratory)^22^ |
| Blood Cholesterol | 6·33 |  |
| Complete blood count | 2·67 |  |
| Urea & Electrolytes | 5·00 |  |
| Liver function test | 4·83 |  |
| Cardiac biomarkers for ACS | 13·57 |  |
| CT/MRI Head | 83·33 |  |
| ECG | 2·00 |  |
| Echocardiogram | 25·00 |  |
| Cost per outpatient visit | 1·80 | WHO-CHOICE: for Myanmar^21^ |
| Cost per inpatient bed day | 8·11 |  |
|  |  |  |

CT- computed tomography, Echo-echocardiogram, MRI- magnetic resonance imaging

**Table S6. Input parameters: cost per year/ per service**

| **Parameters** | **per year/per service (US$)** | **References** |
| --- | --- | --- |
|  |  |  |
| Amlodipine 10 mg | 2·05 | International medical products price guide^20^ |
| Enalapril 10 mg twice | 5·08 |  |
| Atorvastatin 20 mg | 8·06 |  |
| Aspirin 100mg | 2·49 |  |
| Atenolol 50 mg | 2·22 |  |
|  |  |  |
| **Laboratory test for screening** | 7·33 | From private clinic in Myanmar (adjusted to cost of public laboratory)^22^ |
| -Fasting glucose |  |  |
| -Blood cholesterol |  |  |
|  |  |  |
| **Laboratory test for primary prevention** | 19·83 |  |
| -Fasting glucose |  |  |
| -Blood cholesterol |  |  |
| -Complete blood count |  |  |
| -Urea & electrolytes |  |  |
| -Liver function test |  |  |
|  |  |  |
| **ST elevation myocardial infarction (60.60%)** |  | Treatment and outcomes of acute coronary syndrome in India (CREATE): a prospective analysis of registry data^7^ |
| *Percutaneous coronary intervention (18.18%)* | 1466·67 | Package price from private hospital in Myanmar (adjusted to cost of public hospital) |
|  |  |  |
| *Medical treatment with thrombolysis (42.42%)* | 256·54 |  |
| -inpatient bed days |  | WHO-CHOICE: for Myanmar^30^ |
| -CCU costs |  | Estimate |
| - Cardiac biomarkers and other laboratory tests |  | From private clinic in Myanmar (adjusted to cost of public laboratory) |
| -ECG and Echocardiogram |  |  |
| -Medicines |  | International medical products price guide^20^ |
|  |  |  |
| **Non-ST elevation myocardial infarction (39.40%)** |  |  |
| *Medical treatment without thrombolysis* | 221·22 |  |
| -inpatient bed days |  | WHO-CHOICE: for Myanmar^21^ |
| -CCU costs |  | Estimate |
| - Cardiac biomarkers and other laboratory tests |  | From private clinic in Myanmar (adjusted to cost of public laboratory) |
| -ECG and Echocardiogram |  |  |
|  |  |  |
| **Average cost for acute coronary syndrome** | 462·62 |  |
|  |  |  |
| **Stroke- Medical and supportive treatment cost** | 439·96 | No Mechanical Thrombectomy, Negligible number of Thrombolysis^8,9^ |
| -inpatient bed days, extra nursing, and supportive care | | Estimate |
| -laboratory tests |  | From private clinic in Myanmar (adjusted to cost of public laboratory) |
| -CT/MRI Head |  |  |
|  |  |  |
| **Annual average salary** | 4360·00 | For Myanmar^23^ |
|  |  |  |

ACS- Acute coronary syndrome, CCU- Coronary care unit, CT- Computed tomography, CVD- Cardiovascular diseases, MRI- Magnetic resonance imaging, PCI- Percutaneous coronary intervention, US$- United States Dollar

**Table S7. Input parameters: cost for each health state**

| **Health State** | **Description** | **Total Cost US$** |
| --- | --- | --- |
| **For Intervention Arm** |  |  |
| General population with unknown risk | health centre visit once a year + tests for blood sugar and cholesterol | 9·13 |
| 10-19·9% Risk | health centre visit once a year + tests for blood sugar and cholesterol | 9·13 |
| 20-29·9% Risk | health centre visit twice a year + laboratory test annually + medication for 20-29·9% Risk | 38·62 |
| 30-39·9% Risk | health centre visit twice a year + laboratory test annually + medication for >30% Risk | 41·11 |
| >40% Risk | health centre visit twice a year + laboratory test annually + medication for >30% Risk | 41·11 |
| Chronic CVD | health centre visit twice a year + laboratory test annually + medication for secondary prevention | 41·28 |
| **For both Intervention and Non-intervention Arms** | |  |
| ACVD | Calculated based on available treatment options (SA 1) and get the weighted average treatment cost for ACVD using ratio of ACS and stroke (please see table S6 for detailed treatment) | 449·40 |
|  |  |  |

CVD- Cardiovascular diseases, US$- United States Dollar

# Supplementary Appendix 5. Results: probabilistic sensitivity analysis (PSA) and budget impact analysis (BIA)

## PSA results

Figure S1. Cost-effectiveness acceptability curves from societal perspective showing a probability of cost-effectiveness for one- to three-time Myanmar’s GDP per capita

The uncertainty around the estimates was represented in the cost-effectiveness plane illustrated in Figure S2. From the health care provider perspective, the simulation points were spread mostly over the NE quadrant — 100% for men and 99% for women — indicating that the Sc-PP-SP intervention was more costly and more effective than base scenario (Figure S2. A and B). From a societal perspective, most of the simulation points are in NE quadrant- 71·73% for men and 77·12% for women- indicating that the Sc-PP-SP strategy was more costly and more effective while some simulation points fell in the SE quadrant- 28·27% for men and 22·88% for women- implying that the strategy was more effective but less costly than base scenario (Figure S2. C and D).

ICER = 4200 US$/QALY

ICER = 1400 US$/QALY

ICER = 4200 US$/QALY

ICER = 1400 US$/QALY

ICER = 1400 US$/QALY

ICER = 4200 US$/QALY

Figure S2. Cost-effectiveness planes: Screening, primary and secondary prevention Vs. base scenario

## BIA results

The budget impacts and health benefits of the three interventions over the years 1-3 are summarized in Table S10. Among the three interventions, the Sc-PP-SP option would give the most health benefits, reducing 159,000 acute CVD events and preventing 80,000 acute CVD deaths. The cost from a health care provider perspective would be around US$ 441 million. Although the Sc-PP strategy would cost the most, around US$ 443 million, it would generate a lower level of health benefits, preventing around 137 million acute CVD events and 69 million acute CVD deaths, than the level of benefits from the Sc-PP-SP strategy. The SP strategy would cost around US$ 4·7 million; however, it would only prevent 18,000 acute CVD events and save 9,000 CVD deaths, the lowest level of health benefits provided among the three strategies.

**Table S8. BIA results: budget impacts and health benefits for 50% and 75% coverage of screening, primary prevention, and secondary prevention (Sc-PP-SP) program for Year 1**

|  | **50% coverage** | **75% coverage** |
| --- | --- | --- |
|  | **Sc-PP-SP** | **Sc-PP-SP** |
|  |  |  |
| **Budget** |  |  |
| Total intervention related cost | **91,333,684** | **136,801,630** |
| i) Screening | 49,997,244 | 74,995,865 |
| ii) Primary prevention | 40,421,142 | 60,631,713 |
| iii) Secondary prevention | 915,299 | 1,174,052 |
|  |  |  |
| **Cost saving from reduced ACVD**  ACVD treatment cost without intervention | 53,581,979 | 53,581,979 |
| ACVD treatment cost with intervention | 40,964,683 | 34,787,757 |
| Changes in disease related cost | **12,617,295** | **18,794,221** |
|  |  |  |
|  |  |  |
| **Budget impacts** | **78,716,389** | **118,007,409** |
|  |  |  |
| **Health benefits** |  |  |
| Reduced number of ACVD | 28,076 | 41,821 |
| Reduced number of ACVD death | 13,823 | 20,739 |
|  |  |  |

ACVD- acute cardiovascular disease, CVD- Cardiovascular diseases, PP- Primary prevention, Sc- Screening, SP- Secondary prevention

**Table S9. BIA results: eligible population, budget impacts and health benefits for Years 2 and 3.**

|  | **Year 2** | | | **Year 3** | | |
| --- | --- | --- | --- | --- | --- | --- |
|  | **Sc-PP-SP** | **Sc-PP** | **SP** | **Sc-PP-SP** | **Sc-PP** | **SP** |
|  |  |  |  |  |  |  |
| **Eligible population** |  |  |  |  |  |  |
| for screening | 9,297,432 | 9,297,432 |  | 9,748,569 | 9,748,569 |  |
| for primary prevention (25%) | 784,697 | 784,697 |  | 751,802 | 751,802 |  |
| for primary prevention (>=35%) | 1,123,045 | 1,123,045 |  | 1,065,697 | 1,065,697 |  |
| for secondary prevention | 59,779 |  | 105,205 | 85,683 |  | 146,343 |
|  |  |  |  |  |  |  |
| **Budget** |  |  |  |  |  |  |
| Total intervention related cost | **164,318,392** | **161,835,622** | **4,369,400** | **165,864,951** | **162,306,317** | **6,077,958** |
| i) Screening | 84,912,142 | 84,912,142 |  | 89,032,307 | 89,032,307 |  |
| ii) Primary prevention | 76,923,480 | 76,923,480 |  | 73,274,010 | 73,274,010 |  |
| iii) Secondary prevention | 2,482,770 |  | 4,369,400 | 3,558,634 |  | 6,077,958 |
|  |  |  |  |  |  |  |
| **Cost saving from reduced ACVD for specific intervention(s)**  ACVD treatment cost without intervention | 51,223,280 | 47,309,086 | 3,914,195 | 49,539,912 | 44,130,166 | 5,409,746 |
| ACVD treatment cost with intervention | 27,668,516 | 27,016,920 | 1,151,241 | 26,701,586 | 25,773,240 | 1,591,112 |
| Changes in disease related cost | **23,554,765** | **20,292,166** | **2,762,953** | **22,838,326** | **18,356,926** | **3,818,634** |
|  |  |  |  |  |  |  |
|  |  |  |  |  |  |  |
| **Budget impacts** | **140,763,627** | **141,543,456** | **1,606,447** | **143,026,625** | **143,949,391** | **2,259,325** |
|  |  |  |  |  |  |  |
| **Health benefits** |  |  |  |  |  |  |
| Reduced number of ACVD | 52,414 | 45,154 | 6,148 | 50,820 | 40,848 | 8,497 |
| Reduced number of ACVD death | 26,442 | 22,771 | 3,109 | 25,617 | 20,580 | 4,292 |
|  |  |  |  |  |  |  |

ACVD- acute cardiovascular disease, CVD- Cardiovascular diseases, PP- Primary prevention, Sc- Screening, SP- Secondary prevention

**Table S10. BIA results: eligible population, budget impacts and health benefits for total years 1-3.**

|  | **Total Years 1- 3** | | |
| --- | --- | --- | --- |
|  | **Sc-PP-SP** | **Sc-PP** | **SP** |
|  |  |  |  |
| **Eligible population** |  |  |  |
| for screening | 29,994,871 | 29,994,871 |  |
| for primary prevention (25%) | 2,356,605 | 2,356,605 |  |
| for primary prevention (>=35%) | 3,373,286 | 3,373,286 |  |
| for secondary prevention | 176,768 |  | 308,699 |
|  |  |  |  |
| **Budget** |  |  |  |
| Total intervention related cost | **512,320,321** | **504,978,709** | **12,820,999** |
| i) Screening | 273,938,936 | 273,938,936 |  |
| ii) Primary prevention | 231,039,774 | 231,039,774 |  |
| iii) Secondary prevention | 7,341,612 |  | 12,820,999 |
|  |  |  |  |
| **Cost saving from reduced ACVD for specific intervention(s)** | 154,345,171 | 142,883,932 | 11,461,238 |
| ACVD treatment cost with intervention | 83,065,730 | 81,142,489 | 3,370,975 |
| Changes in disease related cost | **71,279,441** | **61,741,443** | **8,090,264** |
|  |  |  |  |
|  |  |  |  |
| **Budget impacts** | **441,040,881** | **443,237,266** | **4,730,736** |
|  |  |  |  |
| **Health benefits** |  |  |  |
| Reduced number of ACVD | 158,610 | 137,386 | 18,002 |
| Reduced number of ACVD death | 79,618 | 68,890 | 9,100 |
|  |  |  |  |

ACVD- acute cardiovascular disease, CVD- Cardiovascular diseases, PP- Primary prevention, Sc- Screening, SP- Secondary prevention

# References:

1. Methods for the Economic Evaluation of Health Care Programmes - Research Database, The University of York. https://pure.york.ac.uk/portal/en/publications/methods-for-the-economic-evaluation-of-health-care-programmes(8f69bcee-cdac-44fa-871c-f821470df60a)/export.html.

2. Paulden, M. Calculating and Interpreting ICERs and Net Benefit. *Pharmacoeconomics* **38**, 785–807 (2020).

3. World Health Organization. *Prevention of cardiovascular disease : guidelines for assessment and management of total cardiovascular risk*. https://apps.who.int/iris/handle/10665/43685 (2007).

4. Zaw, K. K., Nwe, N. & Hlaing, S. S. Prevalence of cardiovascular morbidities in Myanmar. *BMC Res Notes* **10**, 99 (2017).

5. The 2014 Myanmar Population and Housing Census | MIMU. http://themimu.info/census-data.

6. NCD Countdown 2030: efficient pathways and strategic investments to accelerate progress towards the Sustainable Development Goal target 3.4 in low-income and middle-income countries. *The Lancet* **399**, 1266–1278 (2022).

7. Xavier, D. *et al.* Treatment and outcomes of acute coronary syndromes in India (CREATE): a prospective analysis of registry data. *Lancet* **371**, 1435–1442 (2008).

8. Venketasubramanian, N., Khine, Y. M., Ohnmar, O., Khin, M. P. P. K. & Win, M. T. Burden of Stroke in Myanmar. *Cerebrovasc Dis Extra* **11**, 49–51 (2021).

9. Yangon hospital helps stroke patients with new treatment. *The Myanmar Times* https://www.mmtimes.com/news/yangon-hospital-helps-stroke-patients-new-treatment.html (2020).

10. Schaufler, T. M. & Wolff, M. Cost effectiveness of preventive screening programmes for type 2 diabetes mellitus in Germany. *Appl Health Econ Health Policy* **8**, 191–202 (2010).

11. Wald, N. J. & Law, M. R. A strategy to reduce cardiovascular disease by more than 80%. *BMJ* **326**, 1419 (2003).

12. MacMahon, S. *et al.* Blood pressure, stroke, and coronary heart disease. Part 1, Prolonged differences in blood pressure: prospective observational studies corrected for the regression dilution bias. *Lancet* **335**, 765–774 (1990).

13. Collins, R. *et al.* Blood pressure, stroke, and coronary heart disease. Part 2, Short-term reductions in blood pressure: overview of randomised drug trials in their epidemiological context. *Lancet* **335**, 827–838 (1990).

14. Gaziano, T. A., Opie, L. H. & Weinstein, M. C. Cardiovascular disease prevention with a multidrug regimen in the developing world: a cost-effectiveness analysis. *Lancet* **368**, 679–686 (2006).

15. Law, M. R., Wald, N. J. & Rudnicka, A. R. Quantifying effect of statins on low density lipoprotein cholesterol, ischaemic heart disease, and stroke: systematic review and meta-analysis. *BMJ* **326**, 1423 (2003).

16. Law, M. R., Wald, N. J., Morris, J. K. & Jordan, R. E. Value of low dose combination treatment with blood pressure lowering drugs: analysis of 354 randomised trials. *BMJ* **326**, 1427 (2003).

17. Govender, R. D., Al-Shamsi, S., Soteriades, E. S. & Regmi, D. Incidence and risk factors for recurrent cardiovascular disease in middle-eastern adults: a retrospective study. *BMC Cardiovascular Disorders* **19**, 253 (2019).

18. GHO | By category | Life tables by country - Myanmar. *WHO* https://apps.who.int/gho/data/view.main.61130?lang=en.

19. Avenir Health. https://avenirhealth.org/software-onehealth.php.

20. International Medical Products Price Guide – International Medical Products Price Guide. https://mshpriceguide.org/en/home/.

21. who-choice-estimates-of-cost-for-inpatient-and-outpatient-health-service-delivery.pdf.

22. Than, T. M. *et al.* Unit cost of healthcare services at 200-bed public hospitals in Myanmar: what plays an important role of hospital budgeting? *BMC Health Serv Res* **17**, 669 (2017).

23. Average Salary in Myanmar 2021 - The Complete Guide. http://www.salaryexplorer.com/salary-survey.php?loc=148&loctype=1.

24. World Development Indicators | DataBank. https://databank.worldbank.org/source/world-development-indicators.

25. Mittmann, N., Trakas, K., Risebrough, N. & Liu, B. A. Utility Scores for Chronic Conditions in a Community-Dwelling Population. *Pharmacoeconomics* **15**, 369–376 (1999).

26. Matza, L. S. *et al.* Acute and chronic impact of cardiovascular events on health state utilities. *BMC Health Services Research* **15**, 173 (2015).

27. Swe, E. E. *et al.* Increasing trends in admissions due to non-communicable diseases over 2012 to 2017: findings from three large cities in Myanmar. *Tropical Medicine and Health* **48**, 24 (2020).

28. CPI Inflation Calculator. https://www.bls.gov/data/inflation_calculator.htm.

29. Reference Exchange Rate. https://forex.cbm.gov.mm/index.php/fxrate.

30. WHO | Cost-effectiveness analysis for health interventions. *WHO* https://www.who.int/heli/economics/costeffanalysis/en/.
